# Global competence of medical students: An assessment scale and preliminary investigation in China

**DOI:** 10.1371/journal.pone.0279190

**Published:** 2023-01-12

**Authors:** Yue Shi, Huakang Du, Yunying Feng, Yihan Cao, Haiyang Zhang, Yingjing Ding, Yi Zhao, Lihan Zhang, Peifan Li, Sicheng Cai, Tong Li, Naiqian Cui, Haojie Wu, Jun Zhao

**Affiliations:** 1 Department of Ophthalmology, Peking Union Medical College Hospital, Chinese Academy of Medical Sciences & Peking Union Medical College, Beijing, China; 2 Department of Education, Peking Union Medical College Hospital, Chinese Academy of Medical Sciences & Peking Union Medical College, Beijing, China; 3 Department of Radiology, Peking Union Medical College Hospital, Chinese Academy of Medical Sciences & Peking Union Medical College, Beijing, China; 4 Shanghai Jiao Tong University School of Medicine, Shanghai, China; 5 Fudan University Shanghai Medical College, Shanghai, China; 6 Department of Gastrointestinal Surgery, Peking University Cancer Hospital, Beijing, China; 7 West China School of Medicine, Sichuan University, Sichuan, Chengdu, China; 8 ENT Institute and Otorhinolaryngology, Department of Affiliated Eye and ENT Hospital, Fudan University, Shanghai, China; 9 Department of Gastroenterology, Union Hospital, Tongji Medical College, Huazhong University of Science and Technology, Wuhan, China; 10 Zhongshan School of Medicine, Sun Yat-sen University, Guangzhou, China; 11 First Clinical Medical School, Southern Medical University, Guangzhou, China; 12 Capital Institute of Pediatrics, Beijing, China; Ege University Faculty of Medicine, TURKEY

## Abstract

**Introduction:**

The importance of global competence has been acknowledged in medical care as well as medical education. This study aims to develop a scale assessing the global competence of medical students, determine the factor structure and internal consistency of the scale and explore the underlying factors influencing the global competence of Chinese medical students in 8-year programs.

**Methods:**

A questionnaire (Global Competence Assessment Scale for Medical Students, MS-GCAS) was developed, and a cross-sectional multicenter survey was conducted in 1062 medical students from 10 medical schools in China. Questionnaire data were analyzed using exploratory factor analysis and multiple linear regression.

**Results:**

The exploratory factor analysis revealed a three-factor scale. The MS-GCAS has good internal consistency (Cronbach’s alpha = 0.79 to 0.87). In the multivariate regression analyses, medical education stage (*p*<0.05), the frequency of communicating with foreigners (*p*<0.001), multilingual ability (*p*<0.05) and grade level (*p*<0.05) are associated with the MS-GCAS scores.

**Discussion:**

The MS-GCAS has the potential to serve as a tool to measure the global competence of medical students. This three-factor scale can be used by medical education researches to improve future versions. Medical schools should conduct further educational reforms to promote students’ global competence.

## Introduction

In the twenty-first century, health professionals practice medicine in a rapidly globalized world. Medical students today must not only learn to deliver better care for culturally, ethnically and racially diverse populations but also contribute to a more inclusive and sustainable world. “Competence” refers not merely to skills or knowledge, but a multidimensional capacity involving individual skills, knowledge, attitudes, and values. In 2018, the Organization for Economic Cooperation and Development (OECD) added global competence to the Program for International Student Assessment (PISA) test for the first time [1]. Different from the focus and scope of cultural competence [2] and cultural humility [3], global competence emphasizes human rights and sustainable development rather than cultural differences and self-reflection. The definition of global competence from the OECD PISA emphasizes individuals’ capability to examine local, global or intercultural issues; recognize and respect others’ mindsets and conceptions of the world; openly and adequately interact with people who have diverse cultural backgrounds; and take actions for mutual welfare as well as sustainable development.

In the field of medical care and medical education, comprehensive qualities, especially the competence of dealing with people from different cultures, have gained attention. There are different models of global health education sharing the same goal to promote the combination of cultures in clinical practice and research, such as cultural competence and cultural humility [4, 5]. The goal of cultural competence focuses on learning about different cultures, and cultural humility emphasizes an examination of our own beliefs and cultural identities. Apart from developing awareness of cultural differences and critically thinking about their own background, global competence also puts forward the capacity of creating opportunities to have our own voices heard and build a more sustainable development society [1]. Global competence has been considered by the World Federation for Medical Education (WFME) when developing international standards for further medical education improvement [6]. An increasing number of medical schools have recognized the significance of global competence and a new round of general education reform at Harvard Medical School [7] includes the preparation of courses to develop students’ global competence. The PISA approach to assessing global competence is composed of a cognitive test on global understanding and a set of Likert-type scales for 15-year-old students [1]. However, there is no appropriate assessment tool of global competence in medical education at present. Little is known about what factors influence global competence.

At present, medical education in China includes “5+3” model (5 years of undergraduate medical education and 3 years of standardized residensy training), 8-year program and “3+2” model (3 years of junior college and 2 years of clinical training). The 8-year program is China’s path to training high-level innovative medical professionals. This study aimed to develop a scale to assess medical students’ global competence and to determine the factor structure and internal consistency and to preliminarily explore the underlying factors influencing the global competence of Chinese medical students in 8-year programs.

## Materials and methods

### Study design

This study was a cross-sectional, questionnaire-based survey conducted from December 2018 to January 2019 among eight-year clinical medicine program medical students.

### Study participants

This study involved 1062 eight-year medical program students from 10 medical schools in 8 cities in China: Peking Union Medical College, Peking University Health Science Center, Xiangya School of Medicine (Central South University), Tongji Medical College (Huazhong University of Science and Technology), Southern Medical University, Shanghai Medical College of Fudan University, West China Medical Center (Sichuan University), Shanghai Jiao Tong University School of Medicine, Zhejiang University School of Medicine and Zhongshan School of Medicine. The questionnaires were distributed randomly through the Wenjuanxing platform and all responses were anonymous. All participants gave informed written consent prior to participation. The Chinese version of the questionnaire was used. This study was approved by the Institutional Review Board (IRB) of Peking Union Medical College Hospital (Approval number: S-K644). The authors had no access to information that could identify individual participants during or after data collection.

### Validation and pre‑testing of the questionnaire

The items of the Global Competence Assessment Scale for Medical Students (MS-GCAS) were synthesized based on the result of literature review, mainly the official manuals of *the OECD PISA Global Competence Framework* [1] and relevant studies assessing global health competencies for medical professionals [8, 9]. Then, we randomly selected 20 medical students from different universities to interview them on their understanding of global competence for medical students and the items of MS-GCAS [10]. We collected the information from these 20 students through online questionnaire and optimized the items of MS-GCAS. 6 experts in the fields of medical education, statistics, psychology and international education evaluated face validity, redundancy, and phrasing of the MS-GCAS via an interview or a Web-based questionnaire; some also provided additional qualitative feedback. On the basis of the feedbacks, details of the target group specialization, items elimination and items adaptation were established. Another 20 medical students were then consulted to ensure that the item expressions were unambiguous for medical students in different grades.

In the pre‑testing of the questionnaire, the first draft of the questionnaire was administered to 206 medical students through the Wenjuanxing platform randomly from Peking Union Medical College and Shanghai Jiao Tong University School of Medicine. Based on the feedback from the student interviews and the expert consultations, the eight-year clinical medicine program in China was divided into four stages: premedical courses, basic medicine courses, clinical courses, and clinical clerkship. Equal numbers of students from each stage completed the pilot test. The framework of the initial item pool of the MS-GCAS are depicted in Table S1 in S1 File. Based on the exploratory factor analysis, the items were then further refined or deleted from the questionnaire to obtain better validity and internal consistency (Table S1 in S1 File). The final MS-GCAS questionnaire was composed of 17 structured questions on a 5-point Likert scale (1 = Not at all like me, 5 = Very much like me) or a 4-point Likert scale (1 = I have never learned about this, 4 = I am familiar with this and I would be able to explain this well). The English version of all instruments used in the study are provided in S2 File.

### Statistical analysis

The Shapiro-Wilk test was used to test for normal distribution of the continuous variables. Continuous variables were presented as mean and standard deviations (SD). Category variables were presented as frequencies and percentages. Pairwise item-to-item correlations were performed to determine whether the data was suitable for factor analysis. Principal component analysis with varimax rotation and exploratory factor analysis were used to examine the internal structure validity of the MS-GCAS. The internal consistency reliability was ascertained by Cronbach’s alpha. The correlation coefficients were calculated by Pearson or Spearman correlation analyses. A linear regression model was performed to investigate the associated factors of MS-GCAS scores. Multiple imputation was used to address the presence of missing data. All statistical analyses in this study were performed with SPSS version 25.0 (SPSS Inc. Chicago, IL, USA).

## Results

### Participant profiles

A total of 1062 valid questionnaires were collected. The overall response rate is 84.9% and the school-specific response rate ranged between 75.2% and 93.5%. The number of participants from each college was balanced: the maximum number was 124 (Peking Union Medical College), and the minimum number was 83 (Xiangya School of Medicine, Central South University). Table 1 presents the characteristics of study participants. 459 (43.2%) were male, and 603 (56.8%) were female. Students currently in the premedical course, basic medicine course, clinical skills course, and clinical practice and internship phases constituted 29.8%, 27.5% and 22.5% and 20.2% of the participants, respectively. Students had been participated in various types of exchange programs, including clinical exchange, scientific research exchange, and public health exchange. 14.2% of the students had participated in at least one exchange program, and the most popular exchange destinations included the America, Canada, the Britain and Hong Kong. 40.5% of the students ranked among the top three in academic performance. 16.8% of the students mastered two or more foreign languages. The frequency of communication with foreigners is 10.7% once a year and 38.5% less than once a year.

**Table 1:** Characteristics of the 1062 surveyed medical students.

	Characteristics	Total (%)
**Gender**		
	**Male**	459 (43.2)
	**Female**	603 (56.8)
**Medical education stage**		
	**Premedical courses**	316 (29.8)
	**Basic medicine courses**	292 (27.5)
	**Clinical courses**	239 (22.5)
	**Clinical clerkship**	215 (20.2)
**English level**		
	**CET-6 ≤ 425**	97 (9.1)
	**425 < CET-6 ≤ 550**	522 (49.2)
	**CET-6 > 550**	443 (41.7)
**Number of foreign languages mastered**		
	**1**	883 (83.1)
	**2**	165 (15.5)
	**≥ 3**	14 (1.3)
**Overseas study experience**		
	**None**	911 (85.8)
	**At least once**	151 (14.2)
**Overseas program type (n = 186)**		
	**Clinical exchange**	55 (29.6)
	**Scientific research exchange**	44 (23.7)
	**Public health exchange**	15 (8.1)
	**Course exchange**	31 (16.7)
	**Others**	41 (22.0)
**Overseas program duration**		
	**≤ 1 week**	14 (1.1)
	**≤ 1 month**	74 (6.0)
	**≤ Half a year**	42 (3.4)
	**≤ 1 year**	18 (1.5)
	**> 1 year**	3 (0.2)
**Medical education stage when participate in the overseas program (n = 186)**		
	**Premedical courses**	56 (30.1)
	**Basic medicine courses**	56 (30.1)
	**Clinical courses**	40 (21.5)
	**Clinical clerkship**	34 (18.3)
**Frequency of communication with foreigners**		
	**Once a week**	91 (7.4)
	**Once a month**	168 (13.6)
	**Once every half a year**	193 (15.6)
	**Once a year**	133 (10.7)
	**Less than once a year**	477 (38.5)
**Grade level in school**		
	**Upper 1/3**	430 (40.5)
	**Middle 1/3**	462 (43.5)
	**Lower 1/3**	170 (16.0)

Abbreviations: CET-6, College English Test-6; Grade level, the academic grade ranking.

### Exploratory factor analysis

The detailed results of each MS-GCAS item are displayed in Table 2. An exploratory factor analysis was run on 17 items, and the results are shown in Table 2. The scree plot showed a hitch on the fourth factor; therefore, a three-factor solution was formulated, which explained 57.78% of the total variance. The initial eigenvalues of the components were 5.78, 2.38, and1.66. After rotation, the factors explained 20.84%, 19.46%, and 17.44% of the variance. To better interpret the factors, factor loadings larger than 0.50 are presented in bold.

**Table 2:** The results of exploratory factor analysis of the MS-GCAS.

Item number	Item	N% of respondents							Mean Score	SD		Factor loadings*		
**How well does each of the following statements below describe you? (Score rules)**														
1 point: Not at all like me														
2 point: Not much like me														
3 point: Somewhat like me														
4 point: Mostly like me														
5 point: Very much like me														
		**1**	**2**	**3**		**4**		**5**	**Mean Score**	**SD**		**1**	**2**	**3**
A1	I give space to people from other cultures to express themselves.	0.6	2.5	14.0		45.7		37.2	4.2	0.8		**0.81**	0.17	0.10
A2	I respect people from other cultures as equal human beings.	0.8	2.1	12.3		40.0		44.8	4.3	0.8		**0.76**	0.23	0.05
A3	I believe that there are two sides to every question and try to look at them both.	0.4	2.4	15.3		41.1		40.8	4.2	0.8		**0.75**	0.12	0.11
A4	I try to look at everybody’s side of a disagreement before I make a decision.	0.3	23.0	22.1		47.4		27.2	4.0	0.8		**0.70**	0.12	0.16
A5	If there is a problem with communication, I find ways around it (e.g., by using gestures, re-explaining, and writing).	0.4	2.5	10.7		40.9		45.5	4.3	0.8		**0.67**	0.29	0.02
A6	I deliver high-quality care to all patients, regardless of race, religion and other beliefs or practices, and I am informed by the best available evidence.	0.8	2.2	18.7		46.6		31.7	4.1	0.8		**0.66**	0.26	0.08
B1	I enjoy organizing activities to enable transnational advocacy about health issues.	2.4	13.2	20.7		33.1		30.6	3.8	1.1		0.20	**0.85**	0.09
B2	I enjoy organizing activities to popularize scientific knowledge to improve public health.	2.5	12.5	25.1		35.9		23.9	3.7	1.1		0.22	**0.78**	0.16
B3	I pay attention to solutions of for global governance (e.g., solutions to global health emergencies and the long-term development of medical systems).	3.5	14.4	29.6		30.3		22.2	3.5	1.1		0.12	**0.74**	0.22
B4	I enjoy taking part in activities organized by communities/universities and hospitals to popularize scientific knowledge.	1.4	9.2	26.3		36.3		26.8	3.8	1.0		0.28	**0.74**	0.17
B5	If there is an opportunity, I would love to join in international volunteer activities organized by international organizations.	1.7	9.2	17.5		32.7		38.9	4.0	1.0		0.32	**0.72**	0.04
**How informed are you about the following topics? (Score rules)**														
1 point: I have never learned about this														
2 point: I have learned about this but I would not be able to explain what it is really about														
3 point: I know something about this and could explain the general issue														
4 point: I am familiar with this and I would be able to explain this well														
		1	2		3		4		**Mean Score**		**SD**	1	**2**	3
C1	Describe the impact on health of cross-border flows, including international trade, information and communications technology, and health worker migration.	19.0	53.1		26.0		1.9		2.1		0.7	-0.07	0.20	**0.74**
C2	The impact on health of different political and economic systems.	7.8	45.0		42.3		4.9		2.4		0.7	0.07	0.09	**0.72**
C3	Health-related cultural beliefs of people from different cultural backgrounds.	10.5	50.2		35.4		3.9		2.3		0.7	0.01	0.17	**0.72**
C4	The Chinese healthcare service structure and the undergoing reform of the Chinese medical system.	2.4	42.3		48.7		6.7		2.6		0.6	0.13	0.00	**0.67**
C5	Describe the distribution and variation of major communicable diseases.	2.6	32.5		57.3		7.5		2.7		0.6	0.27	0.03	**0.63**
C6	Explain how global climate change impact human health.	6.3	47.4		42.2		4.1		2.4		0.7	0.10	0.14	**0.61**
**Initial eigenvalues**												5.78	2.38	1.66
**Explained variance after rotation**												20.84	19.46	17.44

* Factor loadings over 0.5 are presented in bold.

Abbreviations: SD, standard deviation.

### Internal consistency

The internal consistency of the 17-item MS-GCAS was high, with the Cronbach’s alphas of the three factors identified of 0.85, 0.87 and 0.79. The correlation coefficients between the three factors are shown in Table 3 (*r from 0*.*261–0*.*520*, *p* < 0.001).

**Table 3:** Internal consistency reliability and correlation between the three factor scores of the MS-GCAS.

MS-GCAS Subscale	Factor1	Factor2	Factor 3	Cronbach’s Alpha
**Factor1**		0.520***	0.261***	0.852
**Factor2**	0.520***		0.339***	0.874
**Factor3**	0.261***	0.339***		0.787

***p<0.001.

Abbreviations: The MS-GCAS, the Global Competence Assessment Scale for Medical Students; Factor1, A1-A6 items of the MS-GCAS; Factor 2, B1-B6 items of the MS-GCAS; Factor3, C1-C5 items of the MS-GCAS.

### Linear regression

The one-way ANOVA analysis of the MS-GCAS score of the different groups is shown in Table S2 in S1 File. In the multivariate regression analyses (Table 4), the students in the clinical clerkship have significantly higher scores of factor 3 than those in the premedical courses (OR = 1.116, 95% CI: 1.037–1.201, *p* = 0.004). The students with the bottom third of the grade level have lower scores of factor 1 than those with the top one-third of the grade level (OR = 0.919, 95% CI: 0.861–0.983, *p* = 0.014). The students who communicate with foreigners less than once a year have significant lower scores of factor 2 (OR = 0.800, 95% CI: 0.715–0.895, *p*<0.001) and factor 3 (OR = 0.776, 95% CI: 0.694–0.868, *p*<0.001) than those communicate with foreigners once a week. Furthermore, the students mastered more than two foreign languages have significantly better scores of factor 3 (OR = 1.075, 95% CI: 1.013–1.140, *p* = 0.017) than those who can only use one foreign language.

**Table 4:** Multivariable linear regression analysis of the MS-GCAS score.

Variable		Factor 1		Factor 2		Factor 3	
		OR (95% CI)	P	OR (95% CI)	P	OR (95% CI)	P
**Gender**	Male	-	-	-	-		
	Female	1.121 (1.055,1.191)	<0.001	1.145 (1.078,1.215)	<0.001	0.969 (0.912,1.028)	0.293
**Medical education stage**	Premedical courses	-	-	-	-	-	-
	Basic medicine courses	0.984 (0.914,1.059)	0.662	0.985 (0.916,1.060)	0.685	1.066 (0.991,1.147)	0.086
	Clinical courses	0.941 (0.874,1.013)	0.108	0.944 (0.877,1.015)	0.122	1.104 (1.026,1.188)	0.008
	Clinical clerkship	0.900 (0.836,0.970)	0.006	0.919 (0.853,0.988)	0.023	1.116 (1.037,1.201)	0.004
**English level**	CET-6 ≤ 425	-	-	-	-	-	-
	425 < CET-6 ≤ 550	0.994 (0.887,1.114)	0.921	0.979 (0.875,1.095)	0.707	1.002 (0.895,1.122)	0.969
	CET-6 > 550	1.078 (0.956,1.214)	0.219	1.063 (0.945,1.195)	0.311	1.003 (0.891,1.129)	0.964
**Number of foreign languages mastered**	1	-	-	-	-	-	-
	2	1.022 (0.963,1.087)	0.465	1.008 (0.950,1.070)	0.785	1.061 (1.000,1.126)	0.052
	≥ 3	1.001 (0.943,1.063)	0.976	1.047 (0.988,1.111)	0.122	1.075 (1.013,1.140)	0.017
**Grade level**	Upper 1/3	-	-	-	-	-	-
	Middle 1/3	0.968 (0.906,1.035)	0.335	1.013 (0.949,1.081)	0.694	0.968 (0.906,1.035)	0.989
	Lower 1/3	0.919 (0.861,0.983)	0.014	0.939 (0.880,1.003)	0.060	0.919 (0.861,0.983)	0.307
**Overseas program duration**	Never	-	-	-	-	-	-
	≤ 1 month	0.969 (0.910,1.033)	0.334	0.983 (0.924,1.046)	0.586	0.976 (0.918,1.039)	0.446
	> 1 month	1.041 (0.976,1.111)	0.215	1.027 (0.965,1.094)	0.401	1.036 (0.972,1.103)	0.280
**Frequency of communication with foreigners**	Once a week	-	-	-	-	-	-
	Once a month	1.013 (0.924,1.112)	0.781	0.960 (0.876,1.050)	0.372	1.010 (0.922,1.106)	0.828
	Once every half a year	1.003 (0.911,1.105)	0.946	0.961 (0.874,1.055)	0.404	0.916 (0.833,1.007)	0.069
	Once a year	0.959 (0.878,1.048)	0.357	0.861 (0.788,0.939)	0.001	0.897 (0.822,0.979)	0.015
	Less than once a year	0.936 (0.835,1.049)	0.258	0.800 (0.715,0.895)	<0.001	0.776 (0.694,0.868)	<0.001
**Adjusted R** ^**2**^		0.040		0.074		0.065	

Abbreviations: OR, odds ratio; CI, confidence interval. For other abbreviations, see the previous table.

Data are presented as mean (SD) or median (25–75% quartile). T

## Discussion

The MS-GCAS is a pioneering tool for the global competence assessment of Chinese 8-year medical students. The present study reveals a three-factor structure of MS-GCAS and presents good internal consistency. The frequency of communicating with foreigners, multilingual ability and grade level in school are associated with MS-GCAS scores.

Our exploratory factor analysis of the 17 items of the MS-GCAS revealed three main factors. The three-factor structure demonstrated three dimensions of global competence as a comprehensive capacity involving skills, knowledge, attitudes, and values. Based on the definition outlines of global competence proposed by OECD [1], factor 1 included 6 items assessing ability to communicate in open, appropriate, and effective ways with people from different cultures or with different beliefs. Factor 2 aligned with ability to take actions for collective well-being and sustainable development, which distinguished global competence from culture related competence [5, 11]. Factor 3 included 6 items assessing ability to examine issues of local, global and cultural significance. The MS-GCAS and its three factors had acceptable to good reliability. Administrators at other medical schools can used the MS-GCAS to shed light on issues related to global competence and further confirmed the exploratory factor analysis results of our study.

In previous studies, language barriers, acculturation strategies and awareness of the importance of global competence were reported to be correlated with students’ global competence development [12–16]. In our study, factor 2 and factor 3 of MS-GCAS are significantly associated with students’ frequency of international communication. Thus, the attempt to incorporate international communication skills into the medical studies may contribute to the development of global competence, especially in the dimension of examining issues of global significance and taking actions for sustainable development. In a study of communication skills training in medical students at Germany, Ede et al. found that just a short intervention can lead to improved communication skills and significantly increase medical students’ confidence in foreign patient interaction [17]. An increasing number of educational institutes are redesigning their curricula to provide more overseas study opportunities, teach students how to stay informed and critically think about important issues related to global health and improve students’ cross-cultural communication skills [18, 19]. Our study indicated a preliminarily 3-factor framework of global competence assessment tool which could be used for evaluation of future global health educational programs. Furthermore, multilingualism was also associated with global competence in our results, whereas the English level assessed by the College English Test-6 (CET-6) was not associated. Language is the foundation of communication and active communication is the foundation of developing a global and intercultural outlook [20]. However, only high scores in CET-6 is unable to fulfill the medical student’s needs for global competence [12]. The importance of multilingual teaching and more practical English training for medical purposes in medical education should not be ignored.

During the COVID-19 pandemic, the disruption of educational activities and travel bans can have a negative impact on exchange programs and international medical conference. However, in response to COVID-19, medical education faculty used technology to transition some curriculum to online formats and implemented team-facilitated and self-directed virtual learning [21, 22]. Program-specific virtual learning platforms have the potential to play an important role in global competence related curriculum innovation and reform when isolation has become a part of everyday life.

MS-GCAS is a novel and simple questionnaire for assessing global competence in medical students. This instrument can be used by medical education researches to improve future versions and evaluate the effect of global health curricula or educational reform in medical education. Medical students could discover their own inadequacies in global competence by self-test and actively interplay between local and global health. Situational questions have been introduced in other surveys, and specific scenes describing cultural issues, behaviors or values have been used to fully evaluate individuals’ capabilities [1]. However, it takes respondents more time to complete these questionnaires, and attention should be paid to the quality of the situation settings. In this regard, the MS-GCAS is rather concise, which allows further adjustment for specific circumstances.

## Limitations

Our study also had several limitations. First, all the participants in this study were from 8-year clinical medicine education programs in China, which limits the generalizability of our findings. Despite this limitation, our results can illustrate the current situation of medical education in China, as eight-year clinical medicine education programs are among the most representative medical school training programs in China. Second, C1-C6 items of the MS-GCAS used a 4-point Likert scale while other items used a 5-point Likert scale. The factor revealed in the exploratory factor analysis may be influenced by the scale of measurement. Third, the adjusted R^2^ in our linear regression models range from 0.040 to 0.074, which indicate that the potential predicting factors of global competence need to be further investigated. Moreover, some items of the MS-GCAS are double barreled (for example, C1, C2 and A4) which may cause confusion to the respondents and need further revision. More evidence of content validity should be computed in the future studies, such as the content validity index [23]. According to the Association for Medical Education in Europe (AMEE) Guide No. 87 [24], more validation studies are also needed to provide additional evidence for reliability and validity of the MS-GCAS and further optimize the items. The MS-GCAS might be used or adapted to create high-quality survey scales fit for global competence evaluation in medical education research.

## Conclusions

The MS-GCAS is a brief self-reported scale to assess global competence in medical students. If further adjusted and validated, the MS-GCAS may become a useful tool for improving global competence training in medical school and for providing guidance on whether educational reforms are effective.

## Supporting information

S1 Checklist STROBE statement—Checklist of items that should be included in reports of *cross-sectional studies*. (DOCX)

S2 Checklist *PLOS ONE* clinical studies checklist. (DOCX)

S1 File Supporting tables. Table S1. The results of exploratory factor analysis of the MS-GCAS in the pilot test. Table S2. One-way ANOVA analysis of the MS-GCAS score.
(DOCX)

S2 File Questionnaire. The translated English version of the questionnaire used in the study.
(DOCX)
