# Supplementary material for: Global competence of medical students: An assessment scale and preliminary investigation in China
Source: PLoS One. 2023 Jan 12;18(1):e0279190. doi: 10.1371/journal.pone.0279190 (PMC9836264; doi:10.1371/journal.pone.0279190)
Supplement: S1 File — Table S1. The results of exploratory factor analysis of the MS-GCAS in the pilot test. Table S2. One-way ANOVA analysis of the MS-GCAS score. (DOCX) [file pone.0279190.s003.docx]

**Table S1:** The results of exploratory factor analysis of the MS-GCAS in the pilot test.

| **Item number** | **Item** | **Factor loadings*** | | | | **Main reason**  **for deletion** |
| --- | --- | --- | --- | --- | --- | --- |
|  |  | **1** | **2** | **3** | **4** |  |
| A1 | I give space to people from other cultures to express themselves. | **0.89** | 0.09 | 0.13 | 0.13 |  |
| A3 | I believe that there are two sides to every question and try to look at them both. | **0.77** | 0.10 | 0.17 | 0.14 |  |
| A4 | I try to look at everybody's side of a disagreement before I make a decision. | **0.76** | 0.14 | 0.21 | 0.01 |  |
| A6 | I deliver high-quality care to all patients, regardless of race, religion and other beliefs or practices, and I am informed by the best available evidence. | **0.69** | 0.21 | -0.06 | 0.06 |  |
| A2 | I respect people from other cultures as equal human beings. | **0.67** | 0.21 | 0.13 | 0.09 |  |
| A5 | If there is a problem with communication, I find ways around it (e.g., by using gestures, re-explaining, and writing). | **0.66** | 0.28 | 0.06 | 0.34 |  |
| Deleted | I understand the impact of personal experience on a person’s health concept and how to get along with the doctor. | **0.61** | 0.42 | 0.12 | -0.04 | Considered somewhat redundant with items within factor 1 |
| B2 | I enjoy organizing activities to popularize scientific knowledge to improve public health. | 0.21 | **0.84** | 0.11 | -0.08 |  |
| B1 | I enjoy organizing activities to enable transnational advocacy about health issues. | 0.17 | **0.81** | 0.08 | 0.18 |  |
| B4 | I enjoy taking part in activities organized by communities/universities and hospitals to popularize scientific knowledge. | 0.28 | **0.79** | 0.10 | -0.11 |  |
| B3 | I pay attention to solutions of global governance (For example solutions to global health emergencies and long-term development of medical system). | 0.11 | **0.75** | 0.19 | 0.17 |  |
| B5 | If there is a chance, I would love to join in international volunteer activities organized by international organizations. | 0.35 | **0.60** | 0.09 | 0.29 |  |
| C2 | The impact on health of different political and economic system (For example the difference between China and America). | 0.10 | 0.13 | **0.78** | 0.02 |  |
| C1 | The impact on health of cross-border flows, including international trade, information and communications technology, and health worker migration. | -0.01 | 0.06 | **0.77** | 0.19 |  |
| C5 | Describe the distribution and variation of major communicable diseases worldwide (For example AIDS and tuberculosis). | 0.11 | -0.01 | **0.73** | 0.15 |  |
| C3 | Health-related cultural beliefs of people from different cultural backgrounds | 0.11 | 0.13 | **0.70** | 0.05 |  |
| C6 | Explain how global climate change impact human health. | 0.06 | 0.12 | **0.70** | 0.14 |  |
| C4 | Chinese healthcare service structure and the undergoing reform of Chinese medical system. | 0.23 | 0.12 | **0.61** | -0.08 |  |
| Deleted | What is your English level? (Reference to CET-6). | 0.09 | -0.04 | 0.15 | **0.71** | **Formed unexplained factor in exploratory factor analysis** |
| Deleted | I can adapt easily to a new culture. | 0.27 | 0.39 | 0.20 | **0.62** |  |
| Deleted | I am capable of overcoming my difficulties in interacting with people from other cultures. | 0.41 | 0.47 | 0.17 | **0.49** |  |
| **Initial eigenvalues** | | 7.36 | 2.62 | 1.79 | 1.09 |  |
| **Explained variance after rotation** | | 20.05 | 17.75 | 16.01 | 7.41 |  |

* Factor loadings over 0.5 are presented in bold.

**Table S2:** One-way ANOVA analysis of the MS-GCAS score.

| **Variable** |  | **Factor 1** | | **Factor 2** | | **Factor 3** | |
| --- | --- | --- | --- | --- | --- | --- | --- |
|  |  | **Mean±SD** | **P** | **Mean±SD** | **P** | **Mean±SD** | **P** |
| **Gender** | Male | 24.377±3.784 | - | 17.943±4.432 | - | 14.706±2.873 |  |
|  | Female | 25.390±3.477 | ＜0.001 | 19.300±4.110 | ＜0.001 | 14.541±2.852 | 0.351 |
| **Medical education stage** | Premedical courses | 25.206±3.829 | - | 19.025±4.528 | - | 14.184±2.880 | - |
|  | Basic medicine courses | 25.223±3.383 | - | 18.928±4.117 | - | 14.548±2.895 | - |
|  | Clinical courses | 24.732±3.459 | - | 18.351±4.021 | - | 14.791±2.649 | - |
|  | Clinical clerkship | 24.456±3.869 | 0.046 | 18.367±4.485 | 0.140 | 15.130±2.861 | 0.002 |
| **English level** | CET-6 ≤ 425 | 24.670±4.368 | - | 18.361±4.562 | - | 14.010±2.935 | - |
|  | 425 < CET-6 ≤ 550 | 24.510±3.603 | - | 18.113±4.335 | - | 14.515±2.706 | - |
|  | CET-6 > 550 | 25.535±3.446 | ＜0.001 | 19.499±4.086 | ＜0.001 | 14.858±2.999 | 0.017 |
| **Number of foreign languages**  **mastered** | 1 | 24.873±3.631 | - | 18.599±4.332 | - | 14.476±2.870 | - |
|  | 2 | 25.358±3.684 | - | 19.133±4.162 | - | 15.164±2.699 | - |
|  | ≥ 3 | 25.143±4.092 | 0.288 | 21.000±3.258 | 0.046 | 16.714±2.701 | ＜0.001 |
| **Grade level** | Upper 1/3 | 25.309±3.378 | - | 18.960±4.241 | - | 14.723±2.873 | - |
|  | Middle 1/3 | 24.926±3.707 | - | 18.868±4.206 | - | 14.647±2.772 | - |
|  | Lower 1/3 | 24.118±3.998 | 0.001 | 17.671±4.586 | 0.002 | 14.235±3.049 | 0.160 |
| **Overseas program duration** | Never | 24.898±3.643 | - | 18.602±4.299 | - | 14.505±2.838 | - |
|  | ≤ 1 month | 24.830±3.818 | - | 19.000±4.518 | - | 14.898±2.759 | - |
|  | > 1 month | 25.905±3.349 | 0.100 | 19.937±3.897 | 0.047 | 15.762±3.083 | 0.002 |
| **Frequency of communication**  **with foreigners** | Once a week | 25.440±4.212 | - | 20.297±3.793 | - | 15.538±3.277 | - |
|  | Once a month | 25.423±3.458 | - | 19.589±4.404 | - | 15.655±2.814 | - |
|  | Once every half a year | 25.233±3.648 | - | 19.513±4.106 | - | 14.876±2.759 | - |
|  | Once a year | 24.707±3.298 | - | 18.030±3.910 | - | 14.602±2.694 | - |
|  | Less than once a year | 24.648±3.665 | 0.050 | 17.971±4.361 | ＜0.001 | 13.964±2.713 | ＜0.001 |

Abbreviations: The MS-GCAS, the Global Competence Assessment Scale for Medical Students; Factor1, A1-A6 items of the MS-GCAS; Factor 2, B1-B6 items of the MS-GCAS; Factor3, C1-C5 items of the MS-GCAS; SD, standard deviation; CET-6, College English Test-6; Grade level, the academic grade ranking.

.
