# Supplementary material for: Global competence of medical students: An assessment scale and preliminary investigation in China
Source: PLoS One. 2023 Jan 12;18(1):e0279190. doi: 10.1371/journal.pone.0279190 (PMC9836264; doi:10.1371/journal.pone.0279190)
Supplement: S2 File — The translated English version of the questionnaire used in the study. (DOCX) [file pone.0279190.s004.docx]

**File S2: Questionnaire Assessing Global Competence**

**Global Competence is composed of 4 dimensions by the definition of** PISA (Program for International Student Assessment). In the latest version of ***PISA 2018 Global Competence Framework***, Global competence is defined as the capacity to examine local, global and intercultural issues, to understand and appreciate the perspectives and world views of others, to engage in open, appropriate and effective interactions with people from different cultures, and to act for collective well-being and sustainable development.

This questionnaire is designed to assess the global competence of the students of 13 medical schools in China. Thank you for your participation. All personal information collected will be kept strictly confidential.

**Part I Basic information**

1. Your gender:

1) Male

2) Female

2. The medical school you currently study in or graduated from

1) Peking Union Medical College

2) Peking University Health Science Center

3) Xiangya School of Medicine, Central South University

4) Tongji Medical College, Huazhong University of Science and Technology

5) Southern Medical University

6) Shanghai Medical College of Fudan University

7) West China Medical Center, Sichuang University

8) Shanghai Jiao Tong University School of Medicine

9) Zhejiang University School of Medicine

10) Zhongshan School of Medicine

3. Which study level are you currently going through:

1) General knowledge course

2) Basic medical knowledge course

3) Clinical skill Course and Clinical Practice

4) Clinical Internship

4. Have you participated in any exchange program before?

1) I have never participated in any exchange program before

2) I have participated in at least one exchange program before

5. How many exchange programs have you participated in?

6. Which country have you exchanged to?

7. How long have you been in this exchange program?

8. Which type of exchange program have you participated in?

1) Clinical exchange

2) Scientific research exchange

3) Public Health exchange

4) Course exchange

5) Others____

9. Which study phase were you in when you participated in this exchange program:

1. General knowledge course

2. Basic medical knowledge course

3.Clinical skill course and clinical practice

4. Clinical internship

10. What is your English level? (Reference to CET-6)

1) 1 point (CET-6: below 360)

2) 2 point（CET-6: 360-400）

3) 3 point（CET-6:400-425）

4) 4 point（CET-6：425-550）

5) 5 point（CET-6：550-600）

6) 6 point（CET-6：above 600）

11. How many foreign languages do you learn? (Requires basic listening, speaking, reading and writing)

*(Please enter a number)* ____________

12. How often do you contact with people from other countries?(Including online and face-to face communication with foreign classmates, patients, friends, coworkers etc.)

1) Once a week

2) Once a month

3) Twice a year

4) Once a year

5) Below once a year

13. Your academic grade ranking:

A. Top 1/3

B. Middle 1/3

C. Later 1/3

**Part II**: The Global Competence Assessment Scale for Medical Students (MS-GCAS)

1. How informed are you about the following topics?

*(Please select one response in each row.)*

|  | I have never learned about this | I have learned about this but I would not be able to explain what it is really about | I know something about this and could explain the general issue | I am familiar with this and I would be able to explain this well |
| --- | --- | --- | --- | --- |
| Describe the distribution and variation of major communicable diseases. |  |  |  |  |
| Explain how global climate change impact human health. |  |  |  |  |
| The Chinese healthcare service structure and the undergoing reform of Chinese medical system. |  |  |  |  |
| The impact on health of different political and economic systems. |  |  |  |  |
| Describe the impact on health of cross-border flows, including international trade, information and communications technology, and health worker migration. |  |  |  |  |
| Health-related cultural beliefs of people from different cultural backgrounds. |  |  |  |  |

2. How well does each of the following statements below describe you? *(Please select one response in each row)*

|  | Very much like me | Mostly like me | Somewhat like me | Not much like me | Not at all like me |
| --- | --- | --- | --- | --- | --- |
| I try to look at everybody's side of a disagreement before I make a decision. |  |  |  |  |  |
| I believe that there are two sides to every question and try to look at them both. |  |  |  |  |  |
| I give space to people from other cultures to express themselves. |  |  |  |  |  |
| I respect people from other cultures as equal human being. |  |  |  |  |  |
| I deliver high-quality care to all patients, regardless of race, religion and other beliefs or practices, and I am informed by the best available evidence. |  |  |  |  |  |
| If there is a problem with communication, I find ways around it (e.g., by using gestures, re-explaining, and writing). |  |  |  |  |  |
| I enjoy taking part in activities organized by communities/universities and hospitals to popularize scientific knowledge. |  |  |  |  |  |
| I enjoy organizing activities to popularize scientific knowledge to improve public health. |  |  |  |  |  |
| If there is an opportunity, I would love to join in international volunteer activities organized by international organizations. |  |  |  |  |  |
| I enjoy organizing activities to enable transnational advocacy about health issues. |  |  |  |  |  |
| I pay attention to solutions of for global governance (e.g., solutions to global health emergencies and the long-term development of medical systems). |  |  |  |  |  |
